# The effect of activated protein C in the experimental disseminated intravascular coagulation model formed by lipopolysaccharide infusion^1^

**DOI:** 10.1590/ACB351102

**Published:** 2020-12-18

**Authors:** Abdullah Öahin, Nazmi Özer

**Affiliations:** I MD, Department of General Surgery, Adana City Training and Research Hospital, Turkey. Acquisition, analysis and interpretation of data; histopathological examinations; critical revision; final approval.; II Associate Professor, Department of General Surgery, University of Health Sciences, Adana City Training and Research Hospital, Turkey. Substantive scientific and intellectual contributions to the study, conception and design, technical procedures, statistics analysis, manuscript preparation and writing, final approval.

**Keywords:** Disseminated Intravascular Coagulation, Lipopolysaccharide Receptors, Fibrinogen, Rats

## Abstract

**Purpose:**

In this experimental study, activated protein C (APC), which has anticoagulant, antithrombotic, profibrinolytic, anti-inflammatory and antiapoptotic properties, was used to prevent coagulopathy in a disseminated intravascular coagulation (DIC) model formatted with lipopolysaccharide (LPS) infusion.

**Methods:**

Twenty-five Wistar albino rats weighting 280 – 320 g each were used. They were randomly divided into three groups: sham, control and study groups. To sham group (n = 5), only normal saline was infused. To control (n = 10) and study groups (n = 10), 30 mg/kg LPS was infused for 4 h from femoral vein. After LPS infusion, 100 µg/kg recombinant APC was given during 4 h in study group. Eight hours later, blood samples were taken from abdominal aorta and the animals sacrificed. From these samples, platelet, prothrombin time (PT), activated partial thromboplastin time (aPTT), fibrinogen and D-dimer levels were studied.

**Results:**

Platelet counts and fibrinogen levels were significantly lower in control and study groups than sham group (p < 0.05). The PT, aPTT and D-dimer levels were significantly higher in control and study groups than in sham group (p < 0.05). When comparing control and study groups, platelet counts were not statistically different (p = 0.36). However, the difference of the fibrinogen levels was significant between these groups (p = 0.0001). While PT and aPTT were longer in the study group compared to the control group (p < 0.05), D-dimer levels were lower in the study group than in control (p = 0.0001).

**Conclusion:**

Use of APC can prevent hypercoagulation and consumption coagulopathy in the DIC as a result of correcting hematological parameters other than prolongation of coagulation time.

## Introduction

Disseminated intravascular coagulation (DIC) is a clinicopathological syndrome in which disseminated intravascular coagulopathy develops with the introduction of procoagulant substances into the circulation in amounts that natural anticoagulant mechanisms cannot neutralize. In DIC, ischemia and tissue necrosis develop due to disseminated fibrin accumulation in the intravascular area. Bleeding occurs as a result of secondary fibrinolysis with excessive use of clotting factors and platelets. Bleeding contributes to the development of multiple organ failure together with hemodynamic and metabolic disorders by jeopardizing to become bloodshot of many organs^1^,^2^.

Disseminated intravascular coagulation is not a disease by itself, but always secondary to an existing disease. It may occur especially after sepsis, various infections, malignancy, obstetric and vascular diseases, severe trauma, toxic and immunological reactions^3^.

Protein C is a vitamin K-dependent zymogen and is converted into its active form, activated protein C (APC), by the thrombin–thrombomodulin complex. The APC provides regulation of thrombin formation by selective destruction of factors Va and VIIIa, limits the inflammatory response and reduces the apoptotic response in endothelial cells due to cytokines. While APC inhibits coagulation and inflammation, it also increases fibrinolytic activity by inactivation of plasminogen activator inhibitor-1 (PAI-1)^4^,^5^.

## Methods

This experimental study was conducted in Erciyes University Faculty of Medicine Hakan Çetinsaya Experimental and Clinical Research Center (DEKAM) between January and February 2007 with the approval of the Ethics Committee (Decision number:01/72).

The study was carried out in accordance with the rules for the care and use of laboratory animals specified in the ARRIVE guideline and the Helsinki declaration.

Twenty-five Wistar albino rats weighing on average 227 g (range 191–310 g) were used in the study. The rats were randomly divided into sham, control and study groups. They were placed in laboratory conditions one week before the experiment. They were kept in polycarbonate cages (20 ± 2 °C room temperature, 50 ± 10% humidity, 12 h light/dark period) and fed with rat food (Purina) in the form of standard dry pellets. Surgical intervention, injections, blood sampling were performed under intramuscular anesthesia of 50 mg/kgketamine and 10 mg/kg xylazine in all rats that were preoperatively fasted for 12 h.

### Experimental groups

Sham group (n = 5): Femoral vein catheterization was performed.

Control group (n = 10): 30 mg/kg lipopolysaccharide (LPS) diluted with 10 mL 0.9% NaCl from the femoral vein catheter was given by continuous infusion for 4 h and DIC was created.

**Figure 1:**
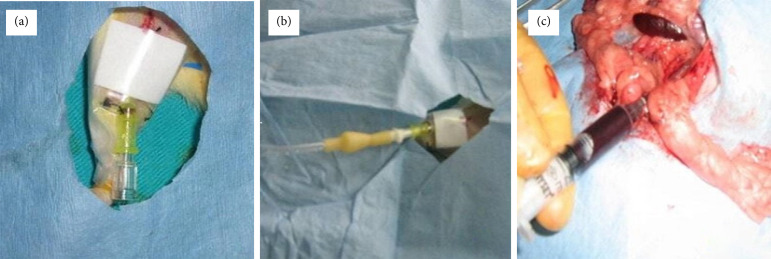
**(a)** Femoral vein catheterization, **(b)** Injection of lipopolysaccharide from the femoral vein, **(c)** Taking blood sample from the aorta.

Study group (n = 10): 30 mg/kg LPS was given by continuous infusion for 4 h through the femoral vein. A blood sample was taken and recombinant APC was given to rats with DIC score 5. Recombinant APC (Drotrecogin alfa [activated]; Xigris, Lilly, Istanbul, Turkey) 100 µg/kg was given by continuous infusion for 4 h through the other femoral vein (Fig. 1).

Eight hours later, blood samples were taken from the abdominal aorta and rats were sacrificed.

### Evaluation parameters

Blood samples were taken from the abdominal aorta by performing midline laparotomy under ketamine hydrochloride anesthesia. Platelet count, prothrombin time (PT), active partial thromboplastin time (aPTT), plasma fibrinogen and D-dimer values were measured from blood samples.

### Statistical analysis

Measurable data were defined as X ± Sd. The conformity of distribution to normal distribution was evaluated by Kolmogorov-Smirnov test. The difference between the groups was examined with the one-way ANOVA test, followed by the Scheffé’s procedure when a significant difference was found. Statistics were performed using the Statistical Package for the Social Sciences (SPSS) for Windows (21.0 version) program, and p < 0.05 values were considered statistically significant.

## Results

 After the DIC was created in this experimental study, the findings obtained from rats that were given recombinant APC (Drotrecogin alpha [activated]) are presented below.

### Thrombocyte (platelet) count

**Figure 2:**
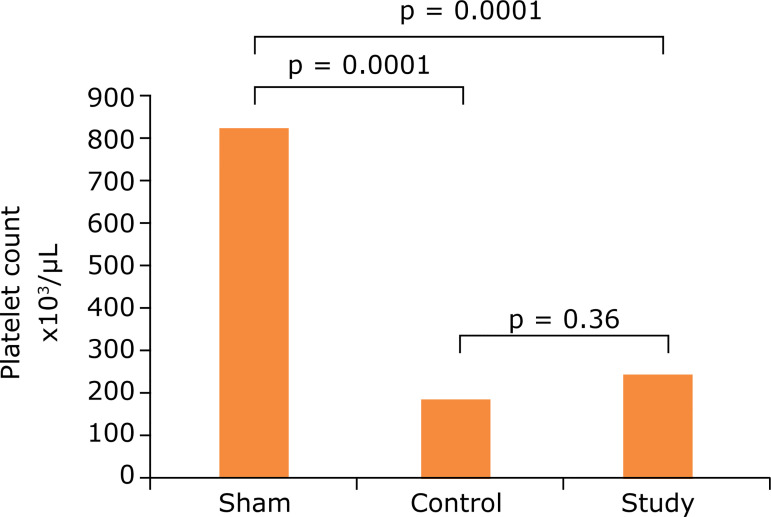
Comparison of the groups according to thrombocyte (platelet) counts.

When the thrombocyte results of all three groups were evaluated, a decrease was observed in the control group (233.9 ± 60.3 × 103/uL) compared to the sham group (859.2 ± 179.7 × 103/uL). The decrease in the control group is statistically significant when compared with the sham group (p = 0.0001). This result showed that thrombocytes were consumed in the DIC. When the working group (295.1 ± 59.37 × 103/uL)and the sham group (859.2 ± 179.7 × 103/uL) were compared, difference was statistically significant (p = 0.0001). When the control and working groups were compared, no statistically significant difference was found (233.9 ± 60.3 × 103/uL and 295.1 ± 59.37 × 103/uL)(p = 0.362) (Table 1, and Fig. 2).

**Table 1:** Comparison of thrombocyte, aPTT, PT, Fibrinogen and D-dimer results between groups.

	Sham group n:5 (X ± Sd)	Control group n:10 (X ± Sd)	Study group n:10 (X ± Sd)	F	P
Thrombocyte (×10^3^/uL)	859.2 ± 179.7^b^ ^c^	233.9 ± 60.3^a^	295.1 ± 59.3^a^	81.4	0.0001
aPTT(s)	24.0 ± 1.0^b^ ^c^	34.3 ± 5.5^a^ ^c^	43.6 ± 5.5^a^ ^b^	26.1	0.0001
PT (s)	13.6 ± 1.1^b^ ^c^	19.5 ± 2.8^a^ ^c^	23.7 ± 3.6^a^ ^b^	18.6	0.0001
Fibrinogen (mg/dL)	233.0 ± 10.0^b^ ^c^	81.4 ± 27.1^a^ ^c^	145.7 ± 33.2^a^ ^b^	50.2	0.0001
D-dimer (µgFEU/mL)	0.3 ± 0.1^b^	5.8 ± 2.2^a^ ^c^	2.0 ± 1.0^b^	25.6	0.0001

a Shows the different groups to sham group.

b Shows the different groups to the control group.

c Shows the different groups to the working group.

One-way ANOVA test: evaluation of differences between groups. Scheffé’s procedure: showing which group is different.

### aPTT

**Figure 3:**
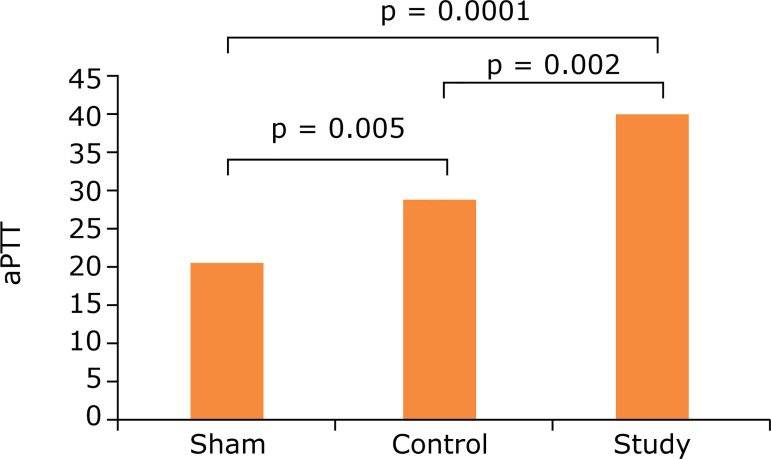
Comparison of the groups accoring to activated Partial Thromboplastin Time (aPTT).

When the aPTT results of all three groups were evaluated, there was a statistically significant difference between all groups (p = 0.0001). The aPTT value, which was 24.0 s on average in the sham group, was prolonged to 34.3 s in the control group. The aPTT were prolonged more in the working group (mean 43.6 s) compared to the control group and were statistically significant (p = 0.002). This elongation was the result of the anticoagulant effect of APC as being in PT. The aPTT was prolonged in the study group compared to the sham group and was statistically significant (to 43.6 from 24.0 s) (p = 0.0001) (Table 1 and Fig. 3).

### PT

**Figure 4:**
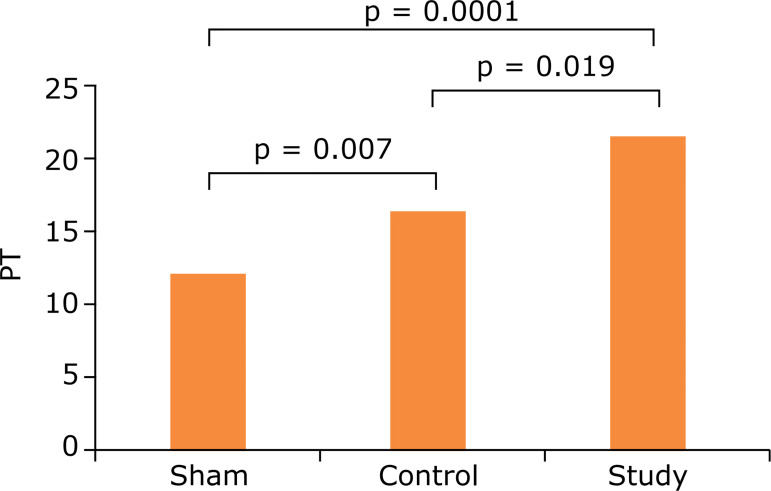
Comparison of groups by prothrombin time (PT).

When the PT results of all three groups were evaluated, the PT value, which was 13.6 s on average in the sham group, was prolonged to 19.5 s in the control group, and this prolongation was statistically significant (p = 0.007). The prolongation of PT was compatible with the DIC diagnostic criteria determined by the International Society of Thrombosis and Haemostasis^6^. The prolongation of PT was higher in the study group (mean 23.7 s) compared to the control and sham groups (13.6, 19.5 and 23.7 s) (p = 0.019, p = 0.0001). The continuation of prolongation of PT in the working group compared to the control group is the result of the anticoagulant effect of APC (Table 1 and Fig. 4).

### Fibrinogen

**Figure 5:**
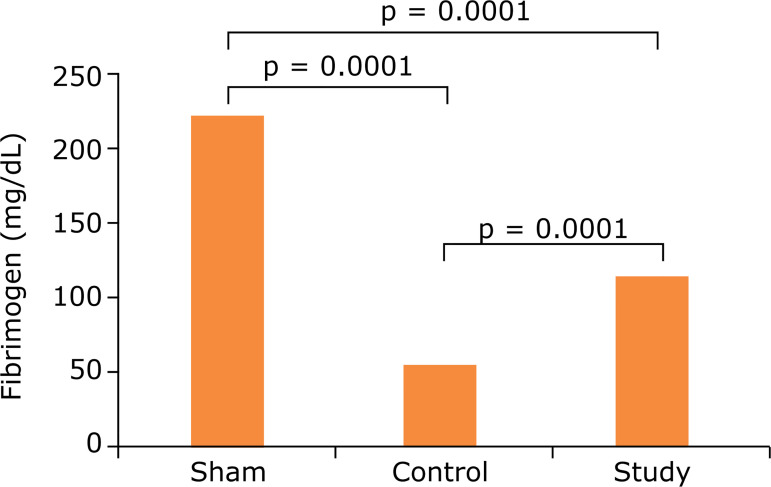
Comparison of the groups according to activated fibrinogen levels.

When the fibrinogen results of all three groups were evaluated, a decrease (81.4 ± 27.1 mg/dL) was observed in the control group compared to the sham group (233.0 ± 10.0 mg/dL) (p = 0.0001). When the control group (81.4 ± 27.1 mg/dL) and the working group were compared, fibrinogen values increased in favor of the working group (145.7 ± 33.2 mg/dL) (p = 0.0001). There was a statistically significant difference between the fibrinogen values of the sham group and the working group (233.0 ± 10.0 mg/dL and 145.7 ± 33.2 mg/dL) (p = 0.0001) (Table 1 and Fig. 5).

### D-dimer

**Figure 6:**
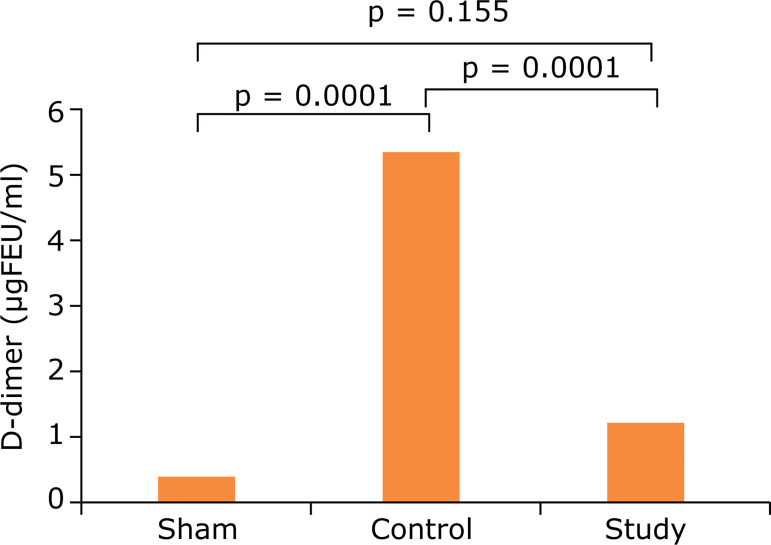
Comparison of the groups according to activated fibrinogen levels.

When the D-dimer results of all three groups were evaluated, an increase was observed in the control group compared to the sham group and this increase was statistically significant (0.3 ± 0.08 µgFEU/mL,5.8 ± 2.2 µg FEU/mL, p = 0.0001). D-dimer was significantly decreased in the working group (2.0 ± 1.0 µg FEU/mL) compared to the control group (5.8 ± 2.2 µg FEU/mL) (p = 0.0001). This decrease may have prevented the formation of new fibrin due to the antithrombotic effect of APC. No statistically significant difference was found between the D-dimer values of the sham group and the working group (0.3 ± 0.08 µg FEU/mL, 2.0 ± 1.0 µg FEU/mL) (p = 0.155) (mg FEU/mL: microgram fibrinogen equivalent unit/milliliter)(Table 1, and Fig. 6).

## Discussion

The DIC model created with LPS is similar to the DIC model that develops in patients with sepsis^7^. In this study, DIC was created by applying LPS infusion (30mg/kg)^8^,^9^.

As a result of the activation of the coagulation system in DIC, coagulation factors and thrombocytes are consumed, protease inhibitors are reduced, and, finally, a clinical situation occurs in which thrombosis and bleeding coexist^10^-^12^.

The primary factor that triggers coagulation is tissue factor (TF). Peripheral blood cells and endothelium do not produce TF under normal conditions. Tissue factor is released as a result of endothelial damage. It is also released into the circulation after trauma, ischemia, severe metabolic stress, tumor, vascular lesions, infections and obstetric problems. Gando *et al*.^13^ found TF in patients with sepsis with a higher value than those who had trauma. The TF-FVIIa unites and triggers the coagulation system, thrombin is formed from prothrombin and fibrin is formed from fibrinogen. Anticoagulant mechanisms limit the activation of the coagulation system without being systemic. When coagulation is systemic and anticoagulant mechanisms are insufficient, bleeding occurs as a result of depletion of thrombocyte and coagulation factors, on the other hand, thrombus occurs due to fibrin accumulation in medium and small vessels, ischemia in the organs due to thrombus and eventually organ failure develops. In DIC, it is aimed to activate the coagulation system starting with TF to suppress the TF-FVIIa combination, and to be stopped from a point by using factor Xa suppressors and thrombin suppressors^14^. For this reason, it is thought that the correction of coagulopathy will have a positive effect on the treatment and, therefore, on morbidity and mortality.

In studies with animals, improvement of DIC resulted in improvement in organ functions and an increase in survival^15^,^16^. In the PROWESS (The Recombinant Human Activated Protein C Worldwide Evaluation in Severe Sepsis) study, it has been shown that drotrecogin alpha (activated) administration, which is a physiological anticoagulant, reduces mortality in septic patients^17^. Therefore, regulation of the coagulation system plays a key role in the planning of sepsis treatment^18^. Disseminated intravascular coagulation is an important factor that can affect the mortality rate in patients with sepsis. In the series of Gogos *et al*.^19^, the mortality rate of patients with sepsis without DIC was 28%, whereas it has been reported as 62% in patients with sepsis accompanied by DIC. Thrombocytopenia is an important finding of acute DIC and is related to bleeding and excessive thrombin production^20^,^21^. Even though thrombocyte production from the bone marrow increases in severe thrombocytopenia, their lifespan is short^21^,^22^. In this study, thrombocyte values decreased in rats with DIC in control and working group. Thrombocyte values were higher in the group given recombinant APC compared to the control group, but it was not statistically significant (p = 0.36). The relative high thrombocyte count may be due to the suppression of thrombin formation by the anticoagulant effect of APC.

Activated protein C shows anticoagulant effect by suppressing APC, FVa and FVIIIa. The anticoagulant effect causes prolongation of PT and aPTT. The low sensitivity of PT and aPTT alone may indicate that it is not safe in the diagnosis of DIC^23^. In this study, prolongation of PT and aPTT was observed in DIC groups. Prothrombin time and aPTT values were significantly longer in the group given APC. This effect is due to the anticoagulant property that occurs as a result of recombinant APC inactivating FVa and FVIIIa. Aoki *et al*.^24^, in a study comparing the effects of APC and heparin on DIC on 132 patients, observed that heparin prolonged aPTT more than APC, decreased the number of thrombocytes and had higher mortality rates.

Fibrinogen, an acute-phase reactant, is used in scoring systems as an indicator of DIC^25^. Fibrinogen levels increase in the early stage of severe infections whereas decrease during the coagulopathy stage. In severe infection and especially in sepsis, a decrease in fibrinogen levels may not be observed unless DIC develops^26^. Matsubara *et al*.^27^, in a multicenter study on 1,103 patients with sepsis, found that patients with DIC have decreased fibrinogen and antithrombin activity, and these levels are significantly associated with an increase in mortality. Wada *et al*.^28^, in a study on 560 patients with DIC found a significant relationship between fibrinogen elevation and organ failure. Secondary fibrinolysis has been observed to be slower in patients with high fibrinogen levels, which has been associated with organ failure and poor survival.In contrast to the study by Wada *et al*.^28^, in this study, subjects who developed DIC had a decrease in fibrinogen levels. With APC treatment, a statistically significant increase was observed in fibrinogen values compared to the control group. The increase in the value of fibrinogen may be due to the anticoagulant and especiallyanti-inflammatory effect of APC. Possibly, it also contributed to preventing the formation of new thrombin. Because thrombin provides the conversion of fibrinogen to fibrin.

Uncontrolled activation of the fibrinolytic system results in increased fibrin degradation products in the plasma. The high level of fibrin degradation products is important in the diagnosis of DIC. One of these products, D-dimer, shows the breakdown of its crosslinked bands and is specific. In DIC, a decrease is observed in the level of fibrinogen, while an increase in D-dimer level is observed. D-dimer occurs through plasmin-mediated degradation of fibrin. High D-dimer level in plasma reflects clot formation^29^,^30^.

In the PROWESS study, an increase in D-dimer levels was observed in 99.7% of the patients^17^. The difference of the PROWESS study from this study is that in the former, a randomized controlled study was conducted on normal patients, and in the latter, it worked on the experimental DIC model with LPS. In this study, D-dimer values were also found to be high. However, a significant decrease was observed in D-dimer values after APC implementation(p = 0.0001). Inhibition of thrombus formation explains the improvement in D-dimer levels due to the improvement in fibrinolysis and organ dysfunction. This result was also consistent with the experimental study conducted by Aoki *et al*.^24^. In the study of Aoki *et al*.^24^, liver and kidney dysfunctions improved with the administration of APC due to DIC. It is possible to attribute the improvement in D-dimer to the suppression of thrombin formation by APC. In addition, prolongation of PT and aPTT, which are markers of anticoagulant effect, can be considered as an indicator of decreased clot formation and consequently reduced destruction.

## Conclusions

Disseminated intravascular coagulation is a condition that occurs secondary to the main pathology. It is a syndrome in which platelet, leukocyte, endothelial cell interactions and activations, various inflammatory cytokines play an important role, and bleeding and thromboembolic events are observed simultaneously in the clinic. The diagnosis should never be made only with the presence of abnormal laboratory tests, the presence of an underlying disease and appropriate clinical findings should be sought. Treatment is to correct the underlying disease and to provide good supportive care.

Activated protein C caused an improvement in coagulation parameters in the DIC model created with LPS, which can be used in DIC treatment and may cause a decrease in mortality and morbidity rates due to DIC. Further clinical studies are needed to use these results in patients with DIC.
